# linc00958/miR-185-5p/RSF-1 modulates cisplatin resistance and angiogenesis through AKT1/GSK3β/VEGFA pathway in cervical cancer

**DOI:** 10.1186/s12958-022-00995-2

**Published:** 2022-09-02

**Authors:** Jing Tian, Lei Cheng, Enqi Kong, Wenjin Gu, Yuanyuan Jiang, Quan Hao, Beihua Kong, Li Sun

**Affiliations:** 1 Department of Gynecological Oncology, Tianjin Medical University Cancer Institute and Hospital, National Clinical Research Center for Cancer, Tianjin, People’s Republic of China; 2 Key Laboratory of Cancer Prevention and Therapy, Tianjin’s Clinical Research Center for Cancer, TianjinTianjin, 300060 China; 3 Department of Gynecology Oncology, Qilu Hospital (Qingdao), Cheeloo College of Medicine, Shandong University, Qingdao, 266035 China; 4 Shandong First Medical University and Shandong Academy of Medical Sciences, Jinan, 250021 China; 5 Department of Gynecological Oncology, Qingdao Central Hospital, The Second Affiliated Hospital of Medical College of Qingdao University, Qingdao, 266042 China; 6 Department of Obstetrics and Gynecology, Cheeloo College of Medicine, Shandong University, Qilu hospital, Jinan, 250012 China

**Keywords:** Cervical cancer, Cisplatin resistance, Linc00958, Angiogenesis

## Abstract

**Background:**

Chemoresistance is one of the major obstacles that lead to poor prognosis in cervical cancer. linc00958 was reported to be an oncogene in cervical cancer. However, its role in mediating chemoresistance remains to be revealed.

**Purpose:**

To explore the regulatory mechanisms of linc00958 in cisplatin-resistant cervical cancer cells and further validate in xenograft mice.

**Methods:**

Online bioinformatic tools were used to conduct the pre-investigation of linc00958/miR-185-5p/RSF-1 and predict the associations between RSF-1 and AKT1/GSK3β/VEGFA in cervical cancer. RT-qPCR measured the RNA expression levels of linc00958/miR-185-5p/RSF-1 in SiHa and SiHa/DDP. Cell survival rates were evaluated by CCK8 methods after cells were exposed to differential concentrations of DDP. Dual-luciferase reporter methods were used to measure luciferase activity. Western blot measured RSF-1 protein and phosphorylated changes of AKT1/GSK3β. Immunofluorescence was employed to observe VEGFA secretion in vitro. Tube formation was applied to evaluate the in-vitro changes of angiogenesis. The SiHa/DDP cells stably transfected with pLKO-sh-NC or pLKO-sh-linc00958 plasmids, were injected into mice, establishing xenograft models. The changes in mice weight and tumor volumes were recorded. H&E staining and Immunohistochemistry (IHC) method was further performed.

**Results:**

linc00958 expression was higher in SiHa/DDP cells. High linc00958 expression was associated with low overall survival. In SiHa/DDP cells linc00958/miR-185-5p/RSF-1 axis inhibited the cellular resistance to cisplatin and suppressed VEGFA and the tube formation through AKT1/GSK3β/VEGFA pathway. The knockdown of linc00958 inhibited RSF-1 and Ki67, curbing tumor growth; it also inhibited VEGFA and CD34, decreasing angiogenesis in mice.

**Conclusion:**

linc00958/miR-185-5p/RSF-1 modulates cisplatin resistance and angiogenesis through AKT1/GSK3β/VEGFA pathway in cervical cancer.

**Supplementary Information:**

The online version contains supplementary material available at 10.1186/s12958-022-00995-2.

## Introduction

Cervical cancer (CC) has one of the highest mortality rates in women among all cancers. Currently, resection surgeries, chemotherapy and radiotherapy are available all over the world. However, the overall and disease-free survival rates stay relatively high, which might be correlated with the undesirable outcomes derived from local resection, chemoresistance, radioresistance, tameless metastasis, etc [1, 2]. Cisplatin is a regular alternative for CC patients at locally advanced stage [3], which in practice is also combined with irradiation therapy or other medications [4, 5]. CC patients tend to gain cisplatin resistance, which in turn poses a challenge to the treatment efficacy [6]. As a result, it is of importance to unveil the potential mechanisms of the chemoresistance in CC. Previous researches explained the causes for drug resistance, which include changes in drug flux, detoxification through DNA repair, cell apoptosis, EMT, DNA methylation, gene profile, signaling pathways, etc [7].

During the past decade, studies identified aunderlying functions of molecules in cisplatin-resistance scenario in therapies for nasopharyngeal carcinoma [8], Osteosarcoma [9], lung cancer [10], breast cancer [11],ovarian cancer [12], cervical cancer [13], among which, noncoding RNAs have emerged as part of the new regulators in cisplatin resistance. lncRNA GAS5, was identified to target miR-21 through PTEN/AKT pathway and knockdown of GAS5 or upregulation of miR-21 added to the cellular resistance to cisplatin in SiHa/DDP in vitro [14].lncRNA CASC2/PTEN, competitively binding to miR-21, impacted on the cisplatin resistance in resistant cells via AKT pathway in CC [15]. The RNA interplay loops, defined as competitive endogenous RNAs (ceRNAs), featuring RNAs competing to bind to a certain miRNA [16, 17]. Linc00958 was revealed earlier in cervical cancer as an oncogenic gene, promoting the cell proliferation and invasion [18], and its downregulation sensitized the cells to radiation through miR-5095/RRM2 [19]. In head and neck carcinoma, linc00958 interacts with MYC and mediates cellular resistance to cisplatin and radiation, suggesting that linc00958 might regulate cisplatin resistance in CC [4].

Further, on online bioinformatic tools starBase and Targetscan, linc00958/RSF-1 were predicted to be targeted by miR-185-5p. Though the role of miR-185-5p in CC hasn’t been revealed yet, miR-185-5p was widely considered to increase cisplatin sensitivity in ovarian, lung and oral cancer cells [20–22]. Previously, RSF-1 was discovered to be related to paclitaxel sensitivity in CC cell line Hela [23]. In addition, downregulation of lncRNA NEAT1 could inhibit cisplatin resistance by suppressing RSF-1 through competitively binding to let-7-5p in nasopharyngeal carcinoma [24]. On the other hand, Gepia database revealed that in CC tumor tissues, RSF-1 expression is positively correlated with VEGFA, AKT1 and GSK3β expression. AKT/ GSK3β pathway was widely involved in cell survival and cisplatin resistance in cancers [25–28]. Vascular endothelial growth factor A(VEGFA) is a key factor associated with angiogenesis in tumors^29^. Taken together, we hypothesized that in CC, linc00958/miR-185-5p/RSF-1 might engage in regulating cisplatin resistance and angiogenesis through AKT1/ GSK3β/ VEGFA pathway. Hence, in this study, we investigated the regulatory effect of linc00958/miR-185-5p/RSF-1 on cisplatin resistance in SiHa/DDP cells and tube formation in vitro; in xenograft mice, we further validated the role of linc00958 in tumor growth and angiogenesis.

## Methods

### Ethical statement

This study has been submitted to and approved by Qingdao University Laboratory Animal Welfare Ethics Committee (Approval No. 202208Balb/C12202306025). No human sample was used in this study. All the cell and animal experiments were performed by strictly following the regulations of Qingdao Central Hospital, The Second Affiliated Hospital of Medical College of Qingdao University.

### Bioinformatic analysis

Gepia (http://gepia.cancer-pku.cn/) was referred to for lncRNAs with differential expression in cervical cancer, in comparison with normal counterparts. After downloading the gene table, we analyzed on Microsoft Excel software to screen the top differential lncRNAs (Table 1). Furthermore, linc00958 expression was also investigated in various types of cancers on Gepia database. Overall survival analysis was conducted on Gepia to examine the correlation between linc00958 expression and overall survival rate, with 15% as the Cutoff-High and 85% as the Cutoff-Low. The linc00958 expression in cervical cancer tissues at early stage or lymph node-positive stage was analyzed in GEO profile (https://www.ncbi.nlm.nih.gov/). RNAinter database (http://www.rnainter.org/search/) was used to predict that miR-185-5p might target at cisplatin and also RSF-1. linc00958 was predicted as a sponge of miR-185-5p in homo sapiens in starBase (http://starbase.sysu.edu.cn/agoClipRNA.php?source=lncRNA), and miR-185-5p targets were predicted on starBase, PITA (https://genie.weizmann.ac.il/pubs/mir07/mir07_data.html) and RNAInter (http://www.rna-society.org/raid/). In addition, the miR-185-5p was predicted to target cisplatin in RNAInter.

**Table 1:** The top differently exprssed lncRNAs in cervical cancer based on Gepia database

GeneSymbol	Gene ID	Log2(Fold change)	Adjusted P value
LINC00925	ENSG00000255571.6	3.691	7.19E-10
LINC00958	ENSG00000251381.6	3.544	3.77E-08
LINC00511	ENSG00000227036.6	3.512	6.06E-22
LINC01133	ENSG00000224259.5	2.673	1.05E-03
LINC00467	ENSG00000153363.12	1.833	7.61E-09
LINC00152	ENSG00000222041.10	1.643	3.48E-10
LINC00960	ENSG00000242516.1	-1.537	1.46E-03
LINC00883	ENSG00000243701.5	-1.592	7.93E-11
LINC00173	ENSG00000196668.3	-1.606	2.37E-09
LINC00092	ENSG00000225194.2	-1.641	5.27E-24
LINC00926	ENSG00000247982.6	-1.663	3.95E-19
LINC00265	ENSG00000188185.11	-1.664	4.97E-13
LINC00115	ENSG00000225880.5	-1.713	1.98E-13
LINC01355	ENSG00000261326.2	-1.732	5.73E-10
LINC00989	ENSG00000250334.5	-1.789	1.80E-56
LINC01125	ENSG00000228486.9	-1.805	3.78E-07
LINC01140	ENSG00000267272.5	-1.85	1.75E-22
LINC01341	ENSG00000227953.6	-1.864	4.20E-08
LINC00893	ENSG00000241769.7	-1.888	2.31E-07
LINC00894	ENSG00000235703.5	-2.033	6.45E-11
LINC00899	ENSG00000231711.2	-2.04	1.20E-16
LINC01139	ENSG00000215808.2	-2.107	2.92E-03
LINC01089	ENSG00000212694.8	-2.287	4.38E-12
LINC01197	ENSG00000248441.6	-2.319	2.38E-58
LINC00890	ENSG00000260802.1	-2.319	2.88E-24
LINC01016	ENSG00000249346.6	-2.345	4.48E-52
LINC00702	ENSG00000233117.2	-2.384	4.22E-19
LINC00908	ENSG00000263812.5	-2.533	3.32E-22
LINC00844	ENSG00000237949.1	-2.642	8.44E-64
LINC-PINT	ENSG00000231721.6	-2.776	1.69E-26
LINC01088	ENSG00000249307.5	-3.456	4.83E-23

### Cell culture

SiHa, SiHa/DDP and HUVECs cells were cultured in Dulbecco’s modified Eagle’s medium (DMEM) with 10% fetal bovine serum (FBS), and 1% penicillin/streptomycin (pen/strep). All cell lines were cultured in a cell incubator with 5% CO2, 99% relative humidity at 37 °C (Thermo Fisher, Shanghai, China).

### Transfection

The overexpressed plasmid of linc00958(pc-linc00958) and RSF-1(pc-RSF-1) was constructed using pcDNA3.1 vector (ThermoFisher, Shanghai, China), with the empty vector as pc-NC. The miR-185-5p mimics, miR-185-5p inhibitor and their control mimics NC and inhibitor NC were provided by GenePharma (Suzhou, China). In brief, 6-well plates were used to seed SiHa and SiHa/DDP cells (5 × 10^5^ cells each well). The 2.5 μg of plasmids pc-linc00958, pc-RSF-1, pc-NC, miR-185-5p mimics and mimics NC were mixed with 4 μl Lipo8000 reagent and added into each well respectively (Beyotime, Shanghai, China) and the transfection was performed according to the producer’s suggestions. After 48 h, cells were measured for transfection effect by RT-qPCR method. The knockdown plasmids of linc00958(sh-linc00958 1#, 2#, 3#), VEGFA(sh-VEGFA-1,2,3) and RSF-1(sh-RSF)were constructed using lentivirus interference vector, pLKO.1-EGFP-Puro (Biofeng, Changsha, China), with the empty vector as sh-NC. The mentioned plasmids were constructed by Tianjin Chuanshi Biotech and were sequenced by Shanghai Biotech(China). The lentivirus plasmids were used to infect the cells to downregulate the expression of linc00958 and RSF-1, together with the packaging vectors pLP1, pLP2, pLP VSV-G. The enhanced green fluorescent protein was observed under an inverted fluorescence microscope after 72 h (Nikon, Japan). Puromycin (1 μg/ml) was added to screen the stable transfected cells. The culture was changed once every three days and the concentration was gradually increased to 12 μg/ml.

### RT-qPCR

Total RNA from SiHa and SiHa/DDP was extracted using Beyozol reagent (Beyotime). We synthesized cDNA by using RT SuperMix for qPCR (Beyotime). Relative RNA levels were measured on a Roche light cycler 96 by using the SYBR qPCR Master Kit with normalization to GAPDH or U6 (Beyotime). The primer sequences used in this study are listed (Table 2). The relative expression of RNAs was calculated using the comparative Ct method.

**Table 2:** primer sequences

LINC00958-Fwd	AGACGCCAGGTAGCTTCTTC
LINC00958-Rev	AGGCTGGAGCCCATCCATTA
RSF1-Fwd	GGCTACACCGGATTGAGACGGATGA
RSF1-Rev	AGGGCTCTGTCCATTGGTTGAAGG
AKT1-Fwd	GGACAAGGACGGGCACATTA
AKT1-Rev	CGACCGCACATCATCTCGTA
GSK3β-Fwd	CCTTGGACTAAGGTCTTCCGA
GSK3β-Rev	ATGGTAGCCAGAGGTGGATTA
VEGFA-Fwd	TTCAAGCCATCCTGTGTGCC
VEGFA-Rev	CACCAACGTACACGCTCCA
GAPDH-Fwd	ACCACAGTCCATGCCATCAC
GAPDH-Rev	TCCACCACCCTGTTGCTGTA
miR-185-5p-RT	GTCGTATCCAGTGCAGGGTCCGAGGTGCACTGGATACGACTCAGGAAC
miR-185-5p-Fwd	TGCGG TGGAGAGAAAGGCAGTTC
U6-RT	GTCGTATCCAGTGCAGGGTCCGAGGTGCACTGGATACGACAAAATATGG
U6-Fwd	TGCGGGTGCTCGCTTCGGCAGC
U6-Reverse	CCAGTGCAGGGTCCGAGGT

### Cell counting Kit-8(CCK8) for cytotoxicity

CCK8 was used to examine cell survival after cisplatin treatment (Bioss, Beijing, China). Following manufacturer’s instructions, cells after transfection were seeded in 96-well plates (8000 cells per well) and incubated for 12 h. Then cells were treated with cisplatin (0, 7.5, 15,30,60, 120, 240ug/ml) for 24 h. Thereafter, 10ul CCK8 solution was added into each well. After 2 h-incubation in the cell incubator, OD values were detected on a microplate reader at 450 nm wavelength (Thermo Fisher, Shanghai, China). Each group was repeated for 3 times.

### Luciferase assays

The luciferase reporter plasmids constructed by inserting the wild-type(wt) linc00958/RSF-1 segments targeted by miR-185-5p or mutant(mt) counterparts onto pGL3 dual luciferase reporter vector (Promega, Shanghai, China). The plasmids included pGL3-linc00958-wt, pGL3-linc00958-mt, pGL3-RSF-1-wt and pGL3-RSF-1-mt. The indicated luciferase plasmids were transfected into SiHa/DDP cells with the miR-185-5p mimics and mimics NC using Lipo8000 kit (Beyotime). After 48 h, luciferase activity was measured on a multi-functional microplate reader after applying Dual Lumi Luciferase reporter Gene Assay kit (Beyotime). The experiments were performed in triplicate.

### Western blot

Protein extracts from cells were prepared using RIPA lysis buffer. Total protein(60 μg) was subjected to SDS-PAGE and transferred to 0.45 μm PVDF membrane (Millipore, USA). Antibodies used in this study were listed and the dilution rates were indicated (Table 3). Membranes were incubated overnight at 4 °C with diluted primary antibody. The membranes were washed using TBST for three times (10 min each time). Then the secondary antibody was used to incubate the membranes for 45 min at room temperature. After washing for another three times, the blotting bands were detected with an ECL kit (Beyotime) on a ECL capture machine (Peiqing, Shanghai, China). The grey values of the bands were analyzed on the analysis software provided by Peiqing.

**Table 3:** Antibodies used in western blot and IHC experiments

Name	Cat number	Dilution rate	Source
Anti-Phospho-Akt1(Ser473)	bsm-52130R	1:200	Bioss
Anti-phospho-AKT (Ser473)	bs-0876R	1:200	Bioss
Anti-AKT	bsm-33278 M	1:200	Bioss
Anti-phospho-GAK3β	bs-2066R	1:200	Bioss
Anti-GAK3β	bs-0023 M	1:200	Bioss
Anti-VEGFA	bs-20393R	1:200	Bioss
Anti-RSF-1	bs-18867R	1:200	Bioss
Anti-Ki67	bs-23103R	1:200	Bioss
Anti-CD34	bs-8996R	1:200	Bioss
Anti-beta-actin	bs-0061R	1: 2000	Bioss
Goat anti-rabbit	bs-0295G	1: 6000	Bioss
Goat anti-mouse	bs-0296G	1: 6000	Bioss

### Immunofluorescence assay (VEGFA)

SiHa/DDP cells were collected in 24-well plates after transfection and washed with PBS. In each well, 200ul paraformaldehyde (4%) was added to fix the cells for 30 min and cold PBS was used to wash the cells for 3 times (5 min each time). Donkey serum (10%) was used to block the cells for 30 min. The primary antibody against VEGFA (bs-4572R, Bioss, Beijing, China) was diluted with 1% donkey serum (1:200) and then added in each well. After overnight incubation at 4℃, the secondary antibody Goat Anti-rabbit IgG H&L/FITC (bs-0295G-FITC, 1:500, Bioss) was added and the cells were incubated in dark for 2 h. Thereafter, cells were washed using PBS for 4 times (5 min each) and DAPI was applied for nucleus staining (Beyotime). The cells were observed under an inverted fluorescence microscope at 100X.

### Tube formation

Matrigel (BD, USA)-coated µ-Slide angiogenesis (ibidi, Germany) was used to analyze tube formation of HUVECs. HUVECs (10^4^) were seeded into each well and 50ul supernatant of SiHa/DDP cells was added. At 8 h, images were taken under inverted microscope. The number of mature tubes was counted in from three different fields. The images were taken under microscope at 100X.

### Xenograft mice model

The cells stably transfected with pLKO-sh-NC and pLKO-sh-linc00958 (differing from the in-vitro group names, sh-NC and sh-linc00958) were collected and adjusted into 5 × 10^7^ cells/ml using preheated PBS. The cells were centrifuged at 1000 rpm for 10 min and then 80ul serum-free medium was added and 100ul cell suspension was injected subcutaneously into the left back of 12 female Balb/C mice (4 weeks old). After 24 days, the tumors are formed, since which, mice weight was measured and tumor volumes were estimated (volume = length*width^2^/2) for the following 28 days. On Day 52, mice were killed and tumors were weighed.

### H&E staining

The tumor tissues derived from the xenograft mice were fixed using 4% paraformaldehyde and dehydrated and embedded using paraffin. Serial sections were cut into 4um per piece. The slides were dewaxed using xylene first and then ethyl alcohol (100%, 95%, 80%, 70%) was used. Harris hematoxylin and eosin(H&E) was used to stain the sections and neutral gum was used to seal the slides. The sections were observed under microscope.

### Immunohistochemistry method

The dewaxed sections were placed into ethyl alcohol (100%, 95%, 85%, 70%, 50% respectively for 40 s each). The 3% H_2_O_2_ was used to incubate the slides for 10 min. Antigen retrieval was conducted using sodium citrate buffer (Bioss). Goat serum was used as the blocking solution (Bioss). The primary antibodies against RSF-1, Ki67, CD34 and VEGFA (1:200, Bioss, Table 3) were added to incubate the sections at 37 °C for 1 h and then PBS was used to wash the slides for three times. The secondary Goat-anti-rabbit antibody (bs-40295G-HRP, 1:3000, Bioss) was applied to incubate the sections for 20 min. DAB kit was purchased from Beyotime and applied to stain the sections. The slides were then placed into hematoxylin for 2 min. The neutral gum was used to block the slides. The sections were observed under microscope.

### Statistical analysis

All the statistical analysis was performed on Excel (Microsoft, USA) and Graphpad9.0 (Graphpad, USA). Student’s t test was conducted where there were only two groups.

One-way ANOVA followed by Tukey’s correction was applied when multiple comparisons were made between groups. Two-way ANOVA and Sidak’s multiple comparison test were performed in cell survival analysis. P < 0.05 was considered significant.

## Results

### Downregulation of linc00958 decreased cisplatin resistance in cervical cancer cells

**Fig. 1:**
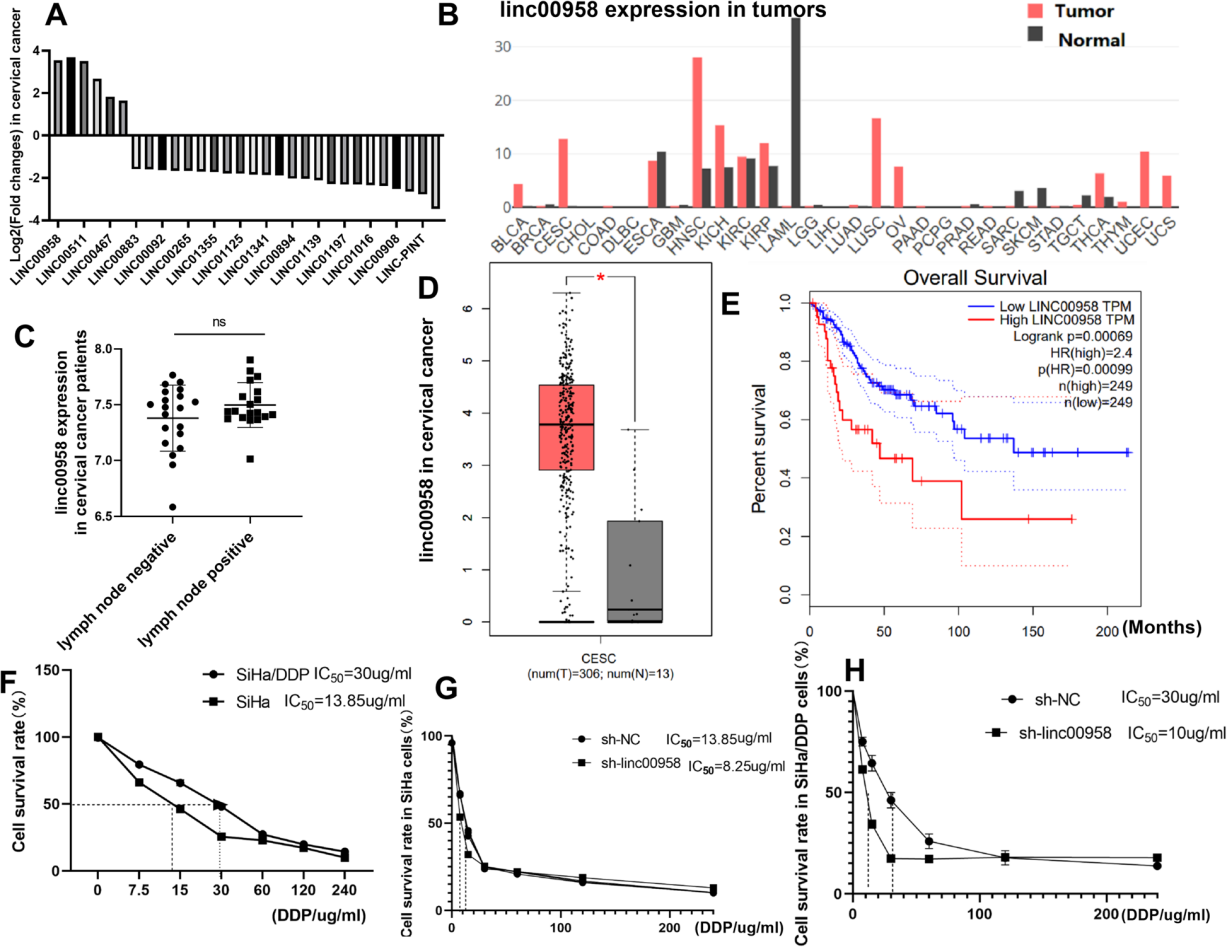
Downregulation of linc00958 decreased cisplatin resistance in cervical cancer cells. **A** Gepia database provided the differentially expressed genes (DEG) in cervical cancer tissues. We screened the top DEGs in Excel and generated the bar graph. **B **Gepia online tool showed the expression of linc00958 in cancers. **C** GEO profile was used to analyze whether linc00958 was associated with the lymph nodes in CC patients. **D**-**E** Differential expression of linc00958 in CC; Overall survival curve in correlation to linc00958 in CC (Gepia). **F**-**H** CCK8 was used to evaluate the cisplatin resistance in SiHa, SiHa/DDP before and after the knockdown of linc00958

Based on Gepia database, linc00958 is among the top upregulated lncRNAs in CC tissues in comparison with the normal counterparts (Fig. 1A&D) and it is also aberrantly upregulated in other cancers, including head and neck squamous cancer, lung cancer, ovarian cancer, etc. (Fig. 1B). Based on GEO profile, linc00958 expression was not associated with the lymph node metastasis in CC (Fig. 1C). Furthermore, low linc00958 was correlated with high overall survival rates among CC patients (Fig. 1E).

In this study, RT-qPCR results showed that linc00958 expression was higher in the cisplatin-resistant cells SiHa/DDP compared to parental SiHa cells (Suppl 1A). After validation of transfection efficiency, by RT-qPCR methods, (Suppl B-F), CCK8 results showed that SiHa/DDP was more reluctant to respond to increasing concentrations of cisplatin with half maximal inhibitory concentration (IC_50_) as 30ug/ml (Fig. 1F). The knockdown of linc00958 in SiHa cells enhanced the cellular sensitivity to cisplatin (Fig. 1G). Further, knockdown of linc00958 in SiHa/DDP cells reduced the IC_50_ of cisplatin, suggesting that the downregulation of linc00958 was correlated with inhibition of cisplatin resistance (Fig. 1H).

### linc00958 mediated the cisplatin resistance via miR-185-5p/RSF-1 axis in cervical cancer cells

**Fig. 2:**
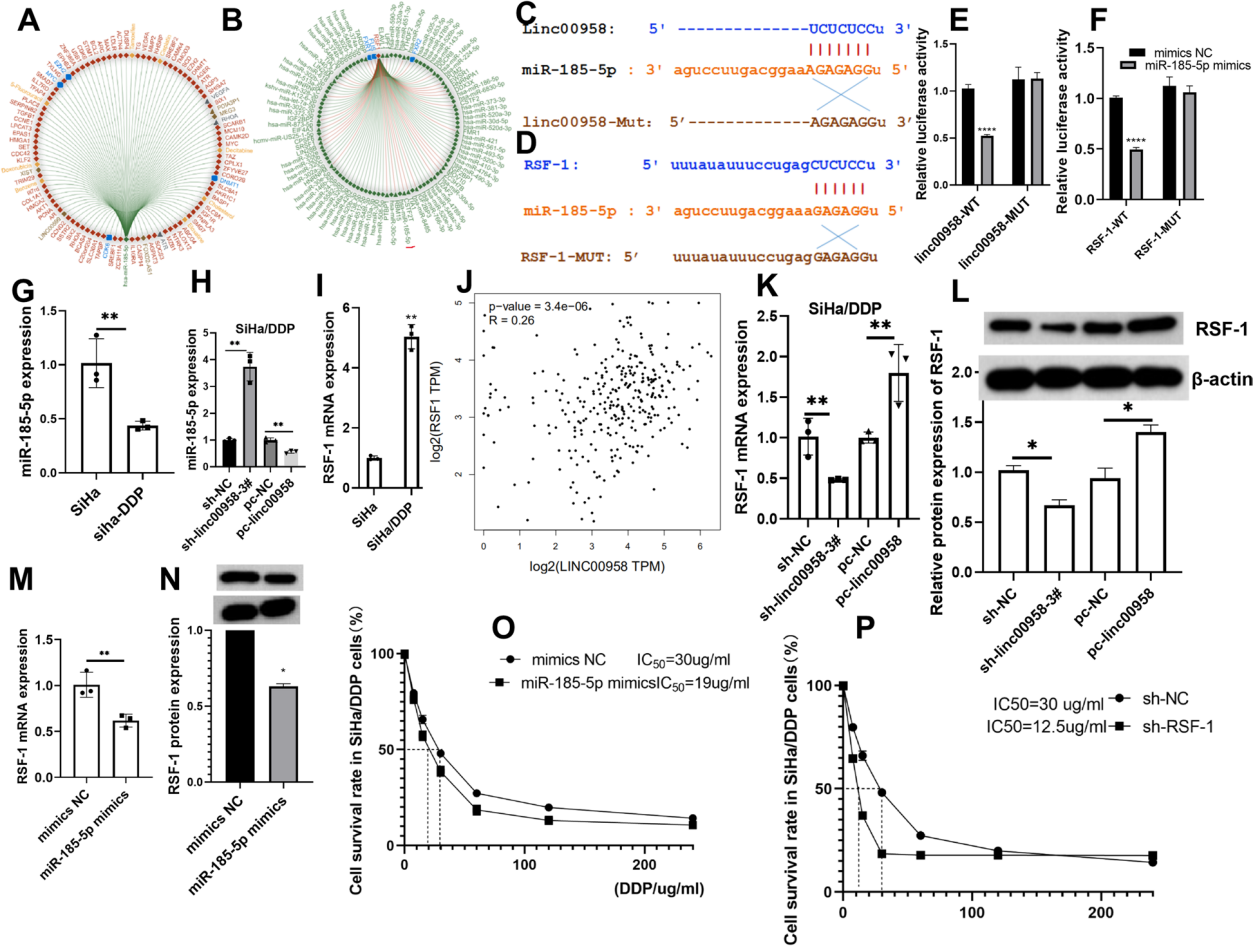
The interactions between linc00958/miR-185-5p/RSF-1 in cervical cancer cells. **A-B** RNAInter was applied to predict cisplatin targets and also miR-185-5p and RSF-1. **C-D** Starbase database provided the putative binding sites between miR-185-5p and RSF-1/linc00958. **E**–**F** Luciferase reporter gene assays. **G**-**I**&**K**-**L** RT-qPCR measured RNA levels of miR-185-5p/RSF-1 in SiHa and SiHa/DDP cells, in SiHa/DDP cells with loss or gain of linc00958. Western blot measured RSF-1 protein expression. **J** Spearman’s correlation analysis between linc00958 and RSF-1 in CC tissues on Gepia. **M**–**N** Impact of miR-185-5p upregulation on RSF-1. **O**-**P** CCK8 evaluated the cisplatin resistance in SiHa/DDP cells with loss of RSF-1 or gain of miR-185-5p. ****P < 0.0001, ***P =  < 0.0002, **P < 0.002, *P < 0.03

RNAInter predicted that miR-185-5p might be related to cisplatin and RSF-1 (Fig. 2A-B). StarBase and PITA databases were applied to further predict the potential binding sites between miR-185-5p and linc00958/RSF-1 (Fig. 2C-D). Luciferase reporter assays in SiHa/DDP cells after co-transfection validated that miR-185-5p targeted RSF-1 and linc00958 sponged miR-185-5p (Fig. 2E-F). RT-qPCR results also revealed that miR-185-5p expression was lower in cisplatin-resistant cell line (Fig. 2G). Furthermore, upregulation of linc00958 could inhibit miR-185-5p in cells (Fig. 2H). On the other hand, RSF-1 mRNA expression was significantly higher in SiHa/DDP cells compared to SiHa (Fig. 2I). Gepia online database showed that linc00958 and RSF-1 were positively correlated in CC tissues (Fig. 2J); In SiHa/DDP cells, knockdown of linc00958 could decrease RSF-1 and linc00958 upregulation could enhance RSF-1 in mRNA and protein levels (Fig. 2K-L). Upregulation of miR-185-5p could decrease mRNA and protein levels of RSF-1 (Fig. 2M-N). Combined together, linc00958 could upregulate RSF-1 and serve as a sponge of miR-185-5p. Furthermore, the upregulation of miR-185-5p or RSF-1 downregulation inhibited the cisplatin resistance in SiHa/DDP cells, suggesting that linc00958 mediated the cisplatin resistance via miR-185-5p/RSF-1 axis in cervical cancer cells (Fig. 2O-P).

### AKT1/GSK3β pathway were modulated by linc00958/miR-185-5p/RSF-1 axis in cervical cancer

**Fig. 3:**
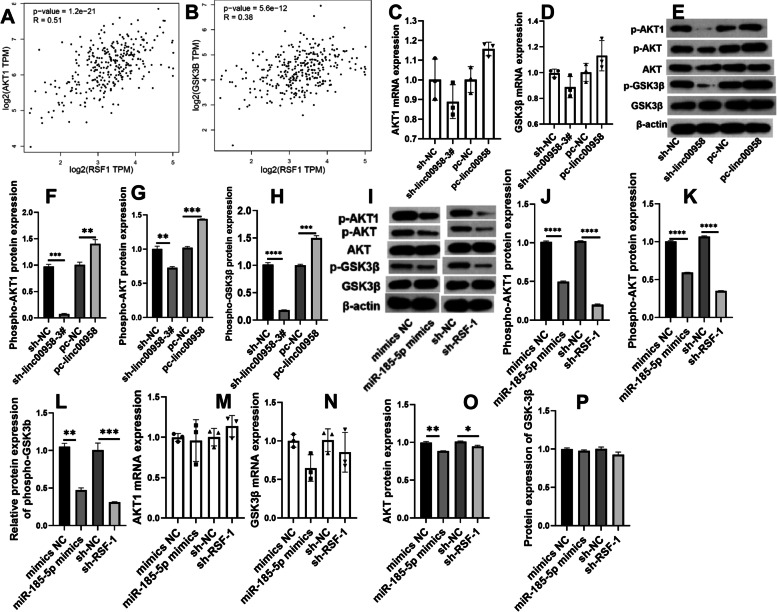
linc00958/miR-185-5p/RSF-1 modulates the AKT1/GSK3β pathway and VEGFA. **A-B** Spearman’s correlation analysis between RSF-1 and AKT1/GSK3β (Gepia). **C-P** Cells were transfected to regulate the expression of linc00958/miR-185-5p/RSF-1. RT-qPCR measured the mRNA expression of AKT1 and GSK3β while western blot detected the protein levels of phospho-AKT1(ser473), phosphor-AKT (ser473), AKT, phosphor-GSK3β and GSK3β. ****P < 0.0001, ***P =  < 0.0002, **P < 0.002, *P < 0.03

RSF1 was correlated with AKT1 and GSK3β in CC tissues according to Spearman’s correlation analysis on Gepia (Fig. 3A-B). RT-qPCR methods validated that in SiHa/DDP cells, upregulation of linc00958 could enhance AKT1 mRNA expression but downregulation of linc00958 didn’t impact on AKT1 mRNA expression significantly (Fig. 3C), while linc00958 regulation didn’t mediate the mRNA of GSK3β in cells (Fig. 3D). However, western blot results supported that phosphorylated AKT1/AKT (ser473) and phosphorylated GSK3β were promoted by linc00958 upregulation and inhibited by downregulation of linc00958 in SiHa/DDP cells (Fig. 3E-H). The efficiency of the downregulated plasmids for RSF-1 were validated using RT-qPCR and western blot (Supp 1F-G). miR-185-5p upregulation or knockdown of RSF-1 inactivated AKT1/GSK3β pathway (Fig. 3I-P). In addition, western blot results confirmed that miR-185-5p inhibitor could upregulate RSF-1 protein and activate AKT1/GSK3β pathway while the knockdown of RSF-1 could partly reverse this (Suppl 2A-G).

### Linc00958/miR-185-5p/RSF-1 axis modulated tube formation through VEGFA pathway

**Fig. 4:**
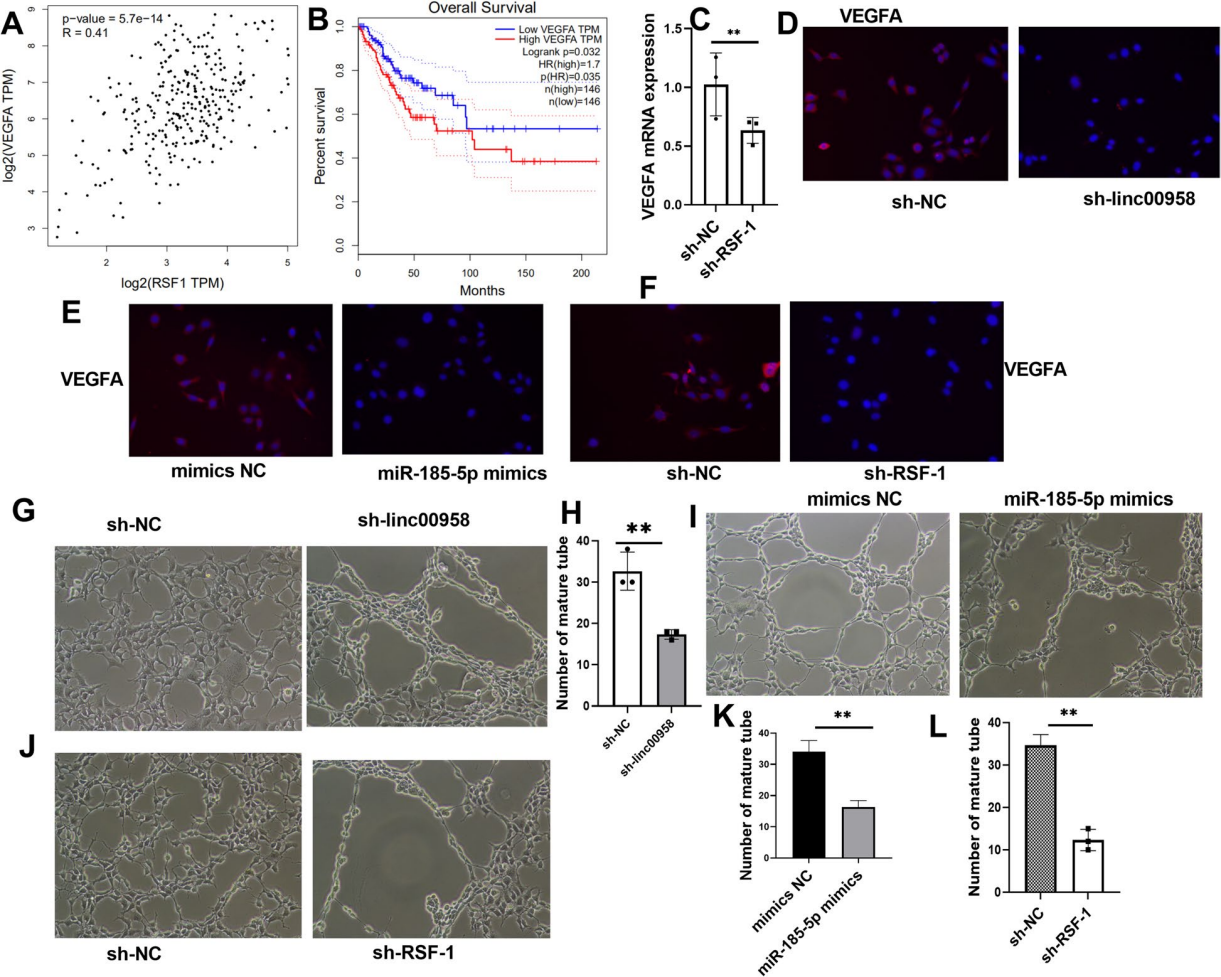
linc00958/miR-185-5p/RSF-1 axis modulated tube formation through VEGFA pathway. **A** Spearman’s correlation analysis between RSF-1 and VEGFA in CC(Gepia). **B** Overall survival in association with VEGFA mRNA expression in CC patients (Gepia). **C-E** Immunofluorescence assays to observe VEGFA secretion in SiHa cells. **F-L **Tube formation assays. **P < 0.002

VEGFA expression was closely correlated with RSF-1 in CC tissues (Fig. 4A). High VEGFA expression was associated with low overall survival rates among CC patients (Fig. 4B). In SiHa/DDP cells, downregulation of RSF-1 could inhibit VEGFA mRNA expression (Fig. 4C). Furthermore, immunofluorescence results showed that VEGFA secretion was inhibited by knockdown of linc00958 and RSF-1 or the overexpression of miR-185-5p in SiHa/DDP cells (Fig. 4D-F). The loss of linc00958/RSF-1 or gain of miR-185-5p in supernatant inhibited the tube formation of HUVECs, blocking angiogenesis in vitro (Fig. 4G-L). The loss of miR-185-5p in supernatant promoted VEGFA secretion and the tube formation while the knockdown of RSF-1 could reverse these (Suppl 2H-J). Furthermore, DDP significantly inhibited VEGFA secretion in SiHa/DDP cells and suppressed the tube formation but miR-185-5p downregulation could reverse the effect of DDP (Suppl 3A-C). After transfection of sh-VEGFA (-1,2,3) in SiHa/DDP cells, it was confirmed that the SiHa/DDP cells in sh-VEGFA-2 group presented the best knockdown effect of VEGFA (Suppl 3D-E). The knockdown of VEGFA in supernatant led to significant inhibition in tube formation (Suppl 3F-G). However, in the cells with VEGFA blocked, neither knockdown of linc00958 nor upregulation of miR-185-5p could further inhibit the tube formation, suggesting that linc00958/miR-185-5p regulated the tube formation through VEGFA pathway (Suppl 3F-G).

### Loss of linc00958 inhibited the tumor growth and angiogenesis in xenograft model

**Fig. 5:**
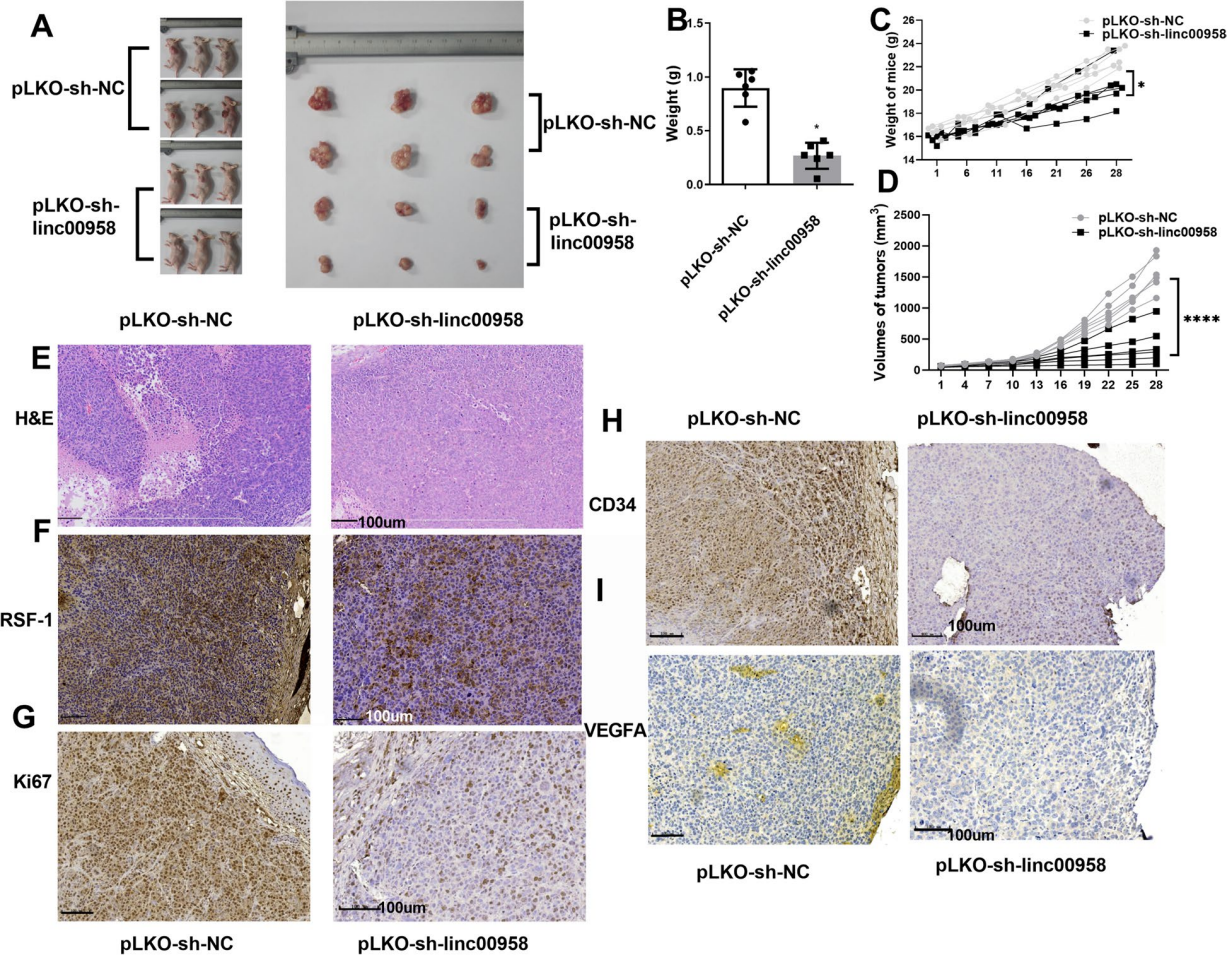
linc00958 downregulation inhibited the tumor growth and angiogenesis in xenograft model. **A** Images of xenograft mice and derived tumors. **B** Tumor weight. **C-D** Weight curve of mice and tumor volume since the first day when tumors could be detected. **E **H&E staining. **F**-**I** IHC measured the changes of RSF-1, Ki67, VEGFA and CD34. Scale: 100um. ****P < 0.0001.*P < 0.03

In vivo, tumor growth was inhibited by loss of linc00958 in mice (Fig. 5A-D). H&E staining showed that downregulation of linc00958 was associated with less severe pathology (Fig. 5E). IHC showed that RSF-1 and the tumor growth biomarker Ki67, were inhibited in the group where linc00958 was knocked down, revealing that linc00958 downregulation in mice could inhibit RSF-1 and Ki67, suppressing the tumor growth (Fig. 5F-G). IHC results also validated that linc00958 downregulation could inhibit the tumor microvessel density marker CD34 and VEGFA in tumors, supporting that the knockdown of linc00958 inhibited the angiogenesis in xenograft model (Fig. 5H-I).

## Discussion

Drug resistance is one of the major concerns leading to bad prognosis in cancer patients [30, 31]. Hence, it is of significance to reveal molecular mechanisms involved. This study focused on the investigation into linc00958 in mediating cisplatin resistance in cervical cancer. linc00958 was validated to upregulate RSF-1 as ceRNA of miR-185-5p in SiHa/DDP cells. Further, linc00958/RSF-1 downregulation or miR-185-5p upregulation could alleviate the cisplatin resistance and tube formation in cervical cancer cells via AKT1/GSK3β/VEGFA pathway. In cisplatin-resistant CC cell-graft mice models, stable knockdown of linc00958 inhibited the tumor growth and angiogenesis through decreasing RSF-1.

Previously, linc00958 downregulation was confirmed to decrease chemo- and radio-resistance in head and neck squamous cell carcinoma in vitro [4]. This study confirmed in CC cell and mice models that linc00958 could inhibit cisplatin resistance. In gastric cancer, miR-185-5p was predicted in the regulation of cisplatin and fluorouracil resistance among gastric cancer patients [32]. However, no former study has been focused on miR-185-5p in cervical cancer yet. In this study, we validated in CC that miR-185-5p upregulation inhibited the cisplatin resistance in SiHa/DDP cells. RSF-1, remodeling and spacing factor 1, engaged in DNA damage repair and cell apoptosis, was reported to reduce cisplatin resistance in malignant melanoma [33] and nasopharyngeal carcinoma [24]. Furthermore, two of our previous in-vitro studies discovered that the RSF-1 downregulation enhanced cell sensitivity to paclitaxel [23] and increased radiosensitivity [34] in CC. In this research, we further discovered that in vitro, depletion of RSF-1 in SiHa/DDP cells could attenuate the cisplatin resistance and tube formation; in xenograft mice, RSF-1 was decreased by the knockdown of linc00958 and associated with the inhibition of tumor growth and angiogenesis.

Interestingly, through online bioinformatics, we noted that RSF-1 was correlated with AKT1, GSK3β and VEGFA expression in CC tissues. Phosphorylation of AKT1 at ser473 is closely involved in cell proliferation, apoptosis and tumor growth [35–37]. The AKT inhibitors including capivasertib, uprosertib and ipatasertib could inhibit the cancer cell survival and drug resistance [38]. Previous research reported that lncRNAs could regulate AKT pathway in cancers [39]. In CC, lncRNA RP1-93H18.6 suppressed the tumorigenesis by inactivating AKT pathway [40]. In this study, we validated that linc00958 could sponge miR-185-5p and upregulate RSF-1 as a ceRNA and linc00958/miR-185-5p/RSF-1 axis could regulate the phosphorylation of AKT1(ser473) and GSK3β, thereby mediating the cell survival and cisplatin resistance in vitro.

VEGF family are essential for blood vessel formation, among which, the VEGFA is a key factor involved with tumor angiogenesis [41]. In CC patients after radiochemotherapy, high VEGFA serum level is correlated with higher rate of early recurrence [42]. In CC patients at advanced stage, the canonical humanized VEGFA monoclonal antibody, bevacizumab, combined with conventional chemotherapy (cisplatin or paclitaxel), could prolong overall survival for 3.7 months [43]. It is of significance to find new molecular mechanisms that regulate VEGFA secretion, which are potential to intervene the CC progression [44]. Previously, depletion of lncRNA HAND2-AS1 could inhibit CC progression by inhibiting VEGFA secretion [45]. Nuclear factor 90(NF90) facilitated angiogenesis in CC by mediating VEGFA [46]. In this study, we revealed that depletion of linc00958/miR-185-5p/RSF-1could inhibit the VEGFA secretion and tube formation in cisplatin-resistant SiHa/DDP cells. In CC mice with knockdown of linc00958, the tumor microvessel density biomarker CD34 and VEGFA were significantly inhibited, revealing that knockdown of linc00958 could attenuate tumor angiogenesis.

## Conclusion

Taken together, this study reported a new regulatory axis linc00958/miR-185-5p/RSF-1 in cisplatin resistance and tube formation in CC via AKT1/GSK3β/VEGFA pathway and knockdown of linc00958 could also inhibit the tumor growth and angiogenesis.

## Supplementary Information

**Additional file 1.**

**Additional file 2.**

**Additional file 3.**

## Data Availability

The analyzed data sets generated during the present study are available from the corresponding author on reasonable request.

## Abbreviations

CC: Cervical cancer
ceRNAs: Competitive endogenous RNAs
DMEM: Dulbecco’s modified Eagle’s medium
FBS: Fetal bovine serum
pen/strep: Penicillin/streptomycin
VEGF: Vascular endothelial growth factor
