# Supplementary figures and images for: linc00958/miR-185-5p/RSF-1 modulates cisplatin resistance and angiogenesis through AKT1/GSK3β/VEGFA pathway in cervical cancer
Source: Reprod Biol Endocrinol. 2022 Sep 2;20:132. doi: 10.1186/s12958-022-00995-2 (PMC9438131; doi:10.1186/s12958-022-00995-2)

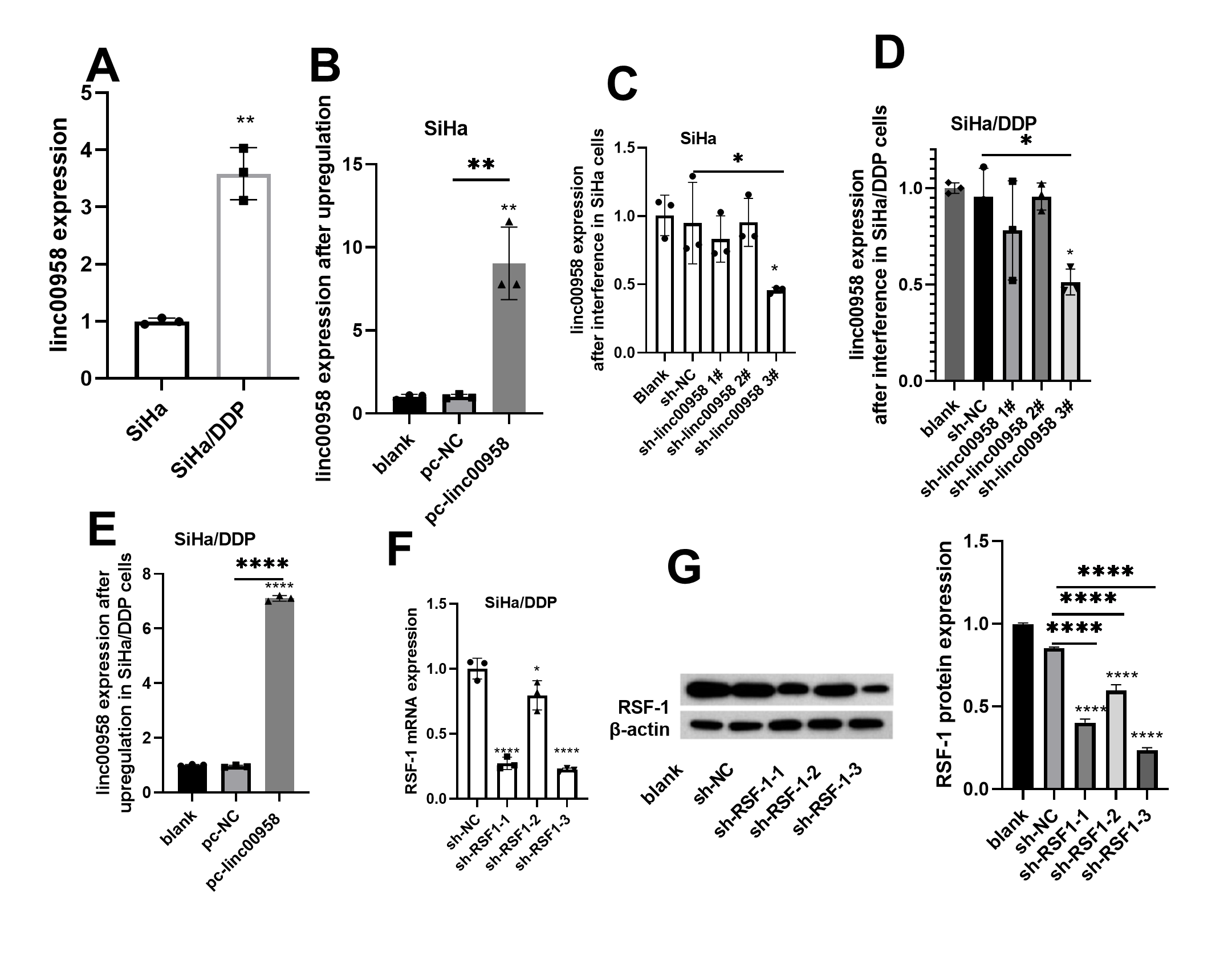

Supplement: Supplementary file 1 — Additional file 1. [file 12958_2022_995_MOESM1_ESM.tif]

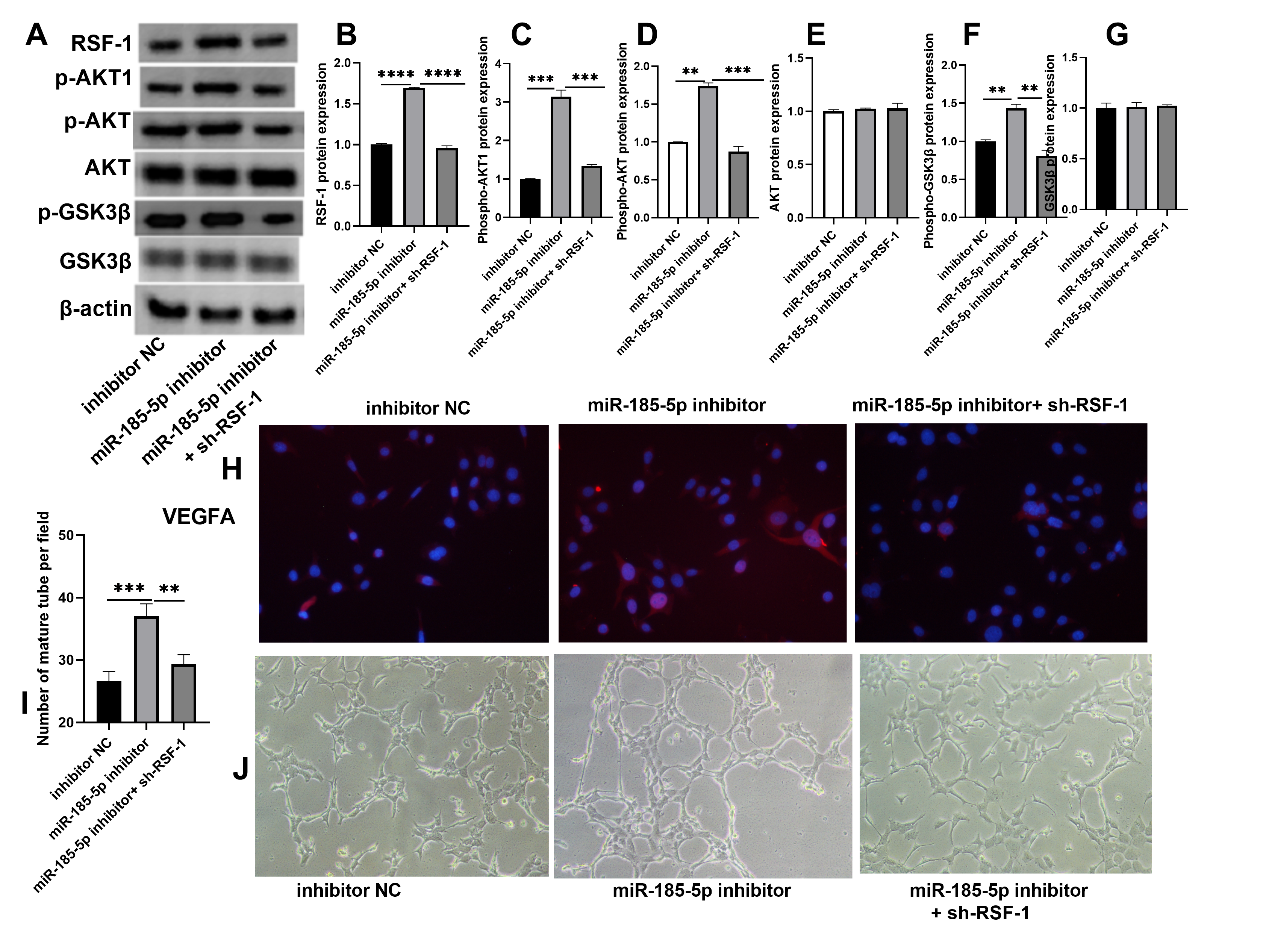

Supplement: Supplementary file 2 — Additional file 2. [file 12958_2022_995_MOESM2_ESM.tif]

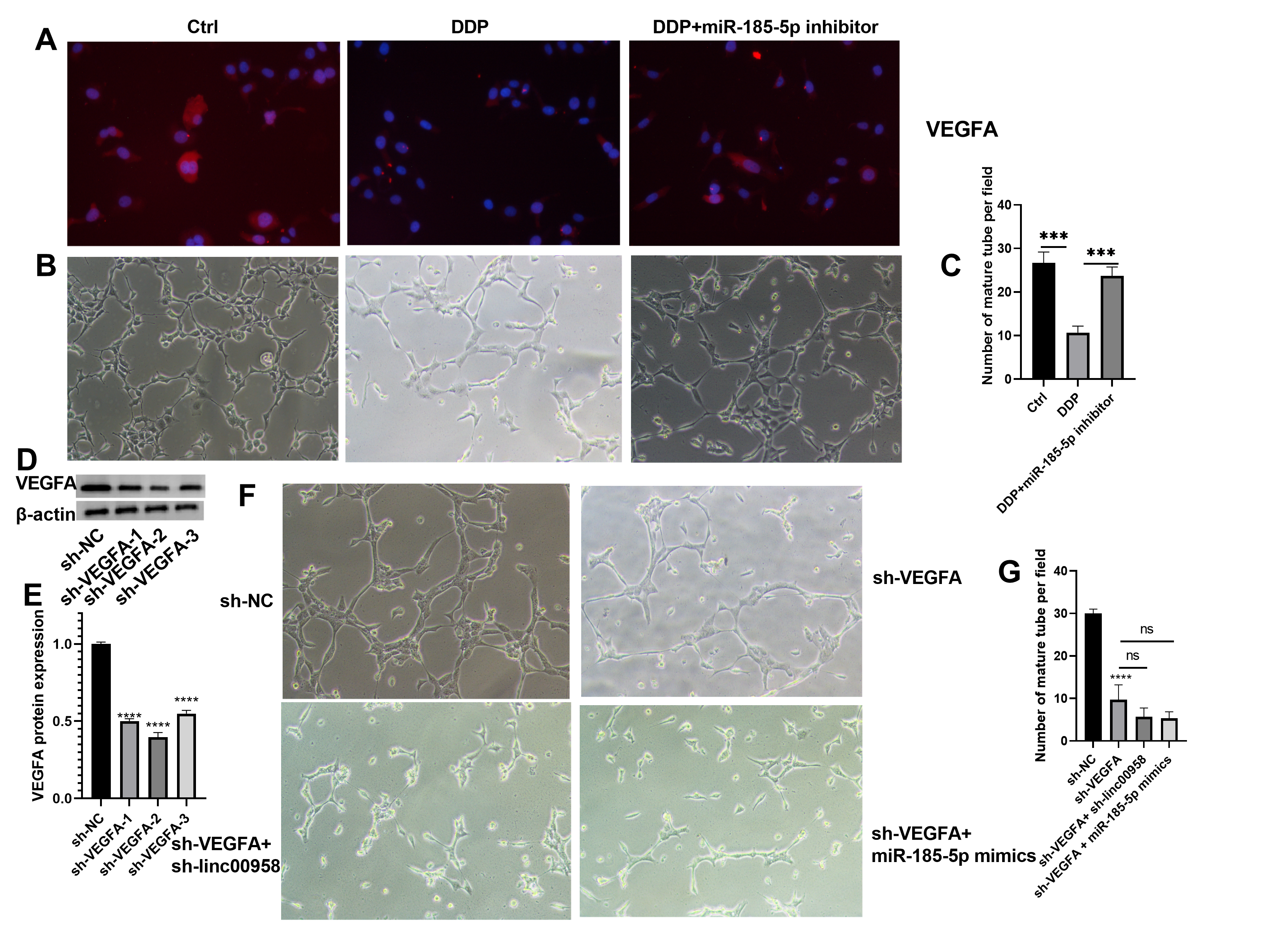

Supplement: Supplementary file 3 — Additional file 3. [file 12958_2022_995_MOESM3_ESM.tif]
